# Moderately Advanced Local Oral Squamous Cell Carcinoma With Significant Tumor Burden

**DOI:** 10.7759/cureus.72285

**Published:** 2024-10-24

**Authors:** Angelo Federico, Maria Miglio, Chadi Faraj

**Affiliations:** 1 General Surgery, Corewell Health, Dearborn, USA

**Keywords:** failure to thrive, jaw reconstruction, oral neoplasm, oral squamous cell carcinoma, orocutaneous fistula, oropharyngeal malignancy

## Abstract

Oral squamous cell carcinoma (OSCC) is a prevalent malignancy with a steadily increasing incidence. Associated risk factors range from tobacco use to the presence of human papillomavirus (HPV) infection. Despite advances in therapeutic options, there have been minimal improvements in the survival rate and percentage of morbidity and mortality. Here, we report the case of a 55-year-old male who presented with failure to thrive secondary to moderately advanced local OSCC, staged T4a N0 M0, and discuss specific considerations regarding prognosis and delayed diagnosis.

## Introduction

Oral squamous cell carcinoma (OSCC) is the eighth leading cause of cancer worldwide and is also the most common malignancy of the head and neck [1]. Annually, about 300,000 cases of oral and oropharyngeal cancer are diagnosed worldwide [2]. Common development sites include the tongue, lips, and floor of the mouth. Risk factors include tobacco use, alcohol consumption, increasing age, human immunodeficiency virus (HIV), and human papillomavirus (HPV). Both tobacco use and alcohol consumption are associated with a six-fold increased risk of developing oral cancer. The combination of tobacco and alcohol use poses a fifteen-fold increased risk of oral cancer [2].

Staging for OSCC is classically performed using the TNM staging system [3]. Prognosis is best when OSCC is diagnosed early, is well-differentiated, and has not yet metastasized. While certain variables, including patient age, comorbidities, tumor size and location, nodal status, oncogene expression, and proliferation markers have been assessed as independent prognostic markers for oral cancer, the most important prognostic factor remains the stage at the time of diagnosis [4]. Delayed diagnosis leads to a delay in treatment and disease progression, which can potentially progress to regional and distant metastasis. Despite the risks associated with delayed diagnosis, most OSCC cases are diagnosed at later stages of the disease. The mortality rate has remained mostly unchanged for decades with an estimated 50% 5-year survival rate, even with advances in surgery and radiotherapy [2]. A delay in diagnosis could be one large contributing factor to the stagnant mortality rate. This case report discusses the workup of a patient with moderately advanced OSCC who experienced a delay in diagnosis.

## Case presentation

A 55-year-old Hispanic male with a known history of T4aN0M0 p16 negative OSCC, with a large tumor invading the tongue, floor of the mouth, mandible, and skin of the face presented to the emergency department with complaints of inability to tolerate oral intake. The patient stated that he had been experiencing new-onset dysphagia with solid foods for approximately two months and difficulty tolerating liquids for approximately three weeks. He noted that more recently when he attempted to consume liquids, approximately half of his oral intake would come out of a pre-existing facial lesion. The patient had no significant past medical or surgical history. Social history was significant for a 15-pack-year history and alcohol intake of an estimated 16 beers per week.

**Figure 1:**
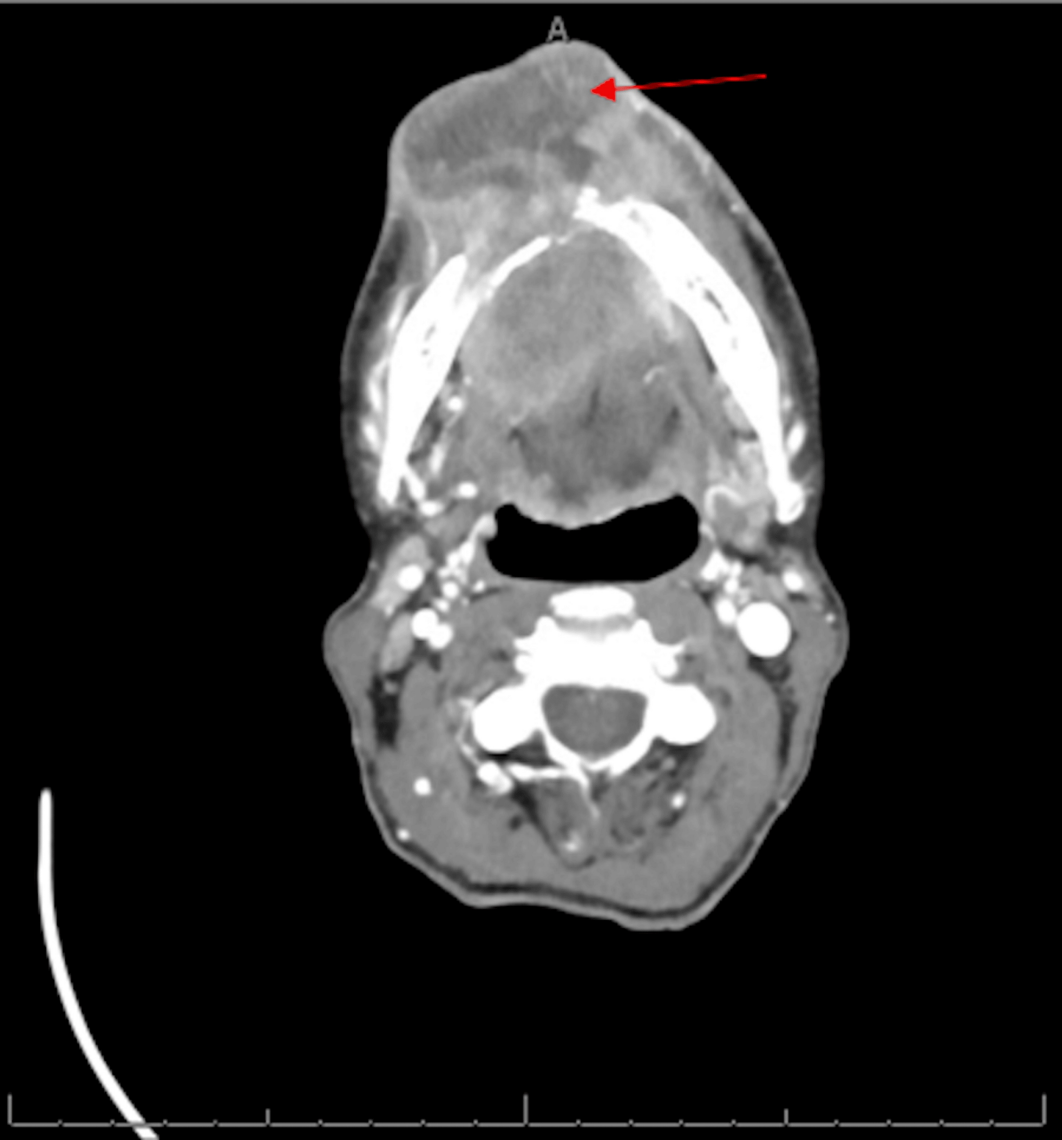
Axial view of the CT soft tissue of the neck showing a large heterogeneous collection (red arrow). It is measuring 6.9 × 6.2 × 3.6 cm, AP × TV × CC dimensions respectively, extending from the anterior soft tissue of the chin into the oral cavity, consistent with severe facial abscess with associated extensive destruction of the mandibular ramus.

The patient was diagnosed with p16 negative invasive moderately to poorly differentiated OSCC just one month before the time of presentation after undergoing an anterior lower gingival buccal sulcus biopsy secondary to concerns for an oral mass and facial swelling. Computed tomography (CT) scan of the soft tissue of the neck at the time of diagnosis demonstrated a large heterogeneous fluid collection extending from the anterior soft tissues of the chin into the oral cavity (Figure 1). Before undergoing the diagnostic biopsy, the patient had previously been seen in the emergency department secondary to concerns for facial swelling but was discharged home on antibiotics as the swelling was presumed to be infectious.

**Figure 2:**
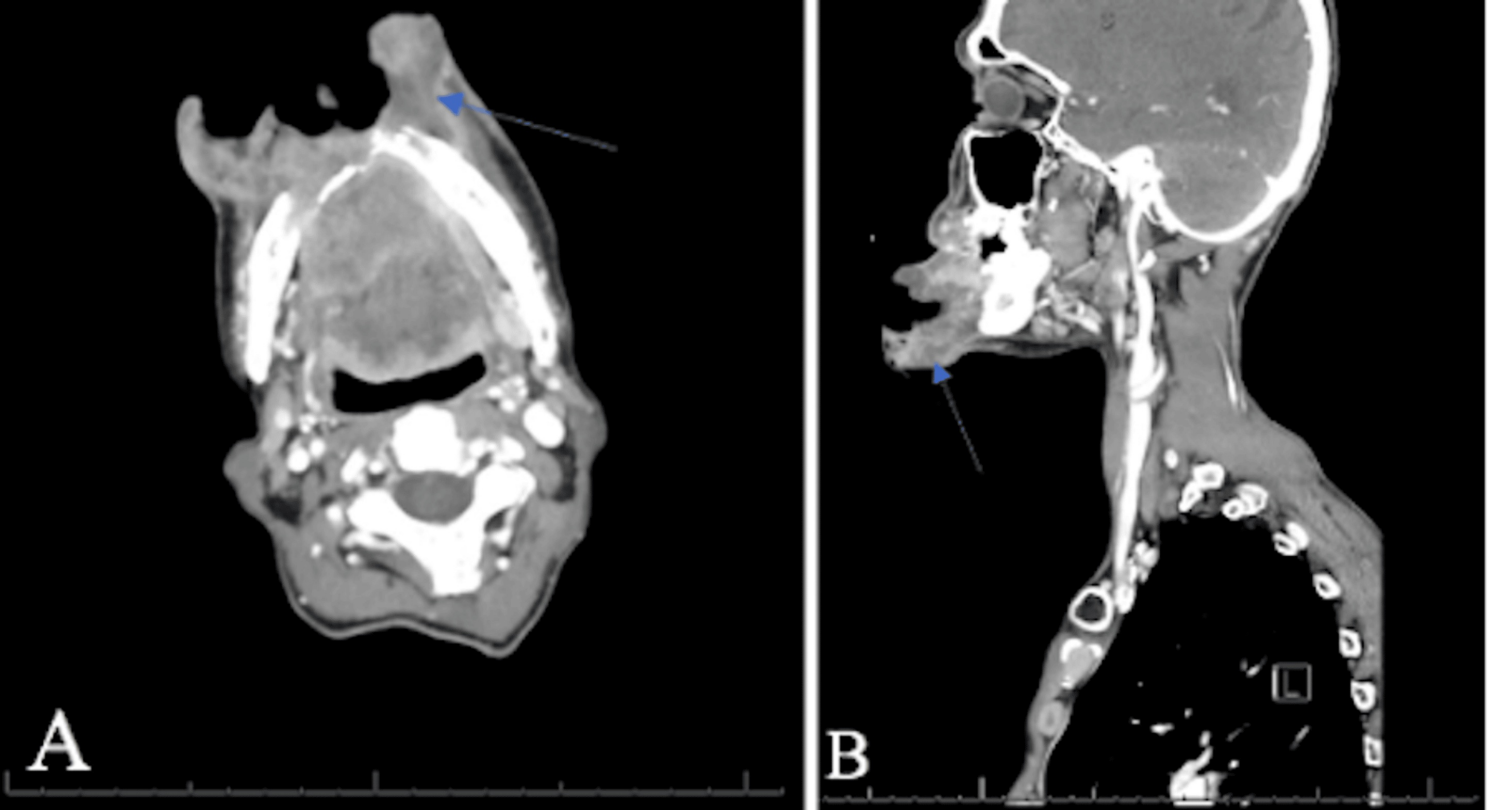
CT soft tissue of the neck demonstrates a large lobulated ulcerating mass. The mass involving the buccal soft tissues extending to the floor of the mouth and gingival surface, with associated destruction of the mandibular ramus (blue arrow). Findings highly suspicious for malignancy, likely squamous cell carcinoma: (A) axial view; (B) sagittal view.

**Figure 3:**
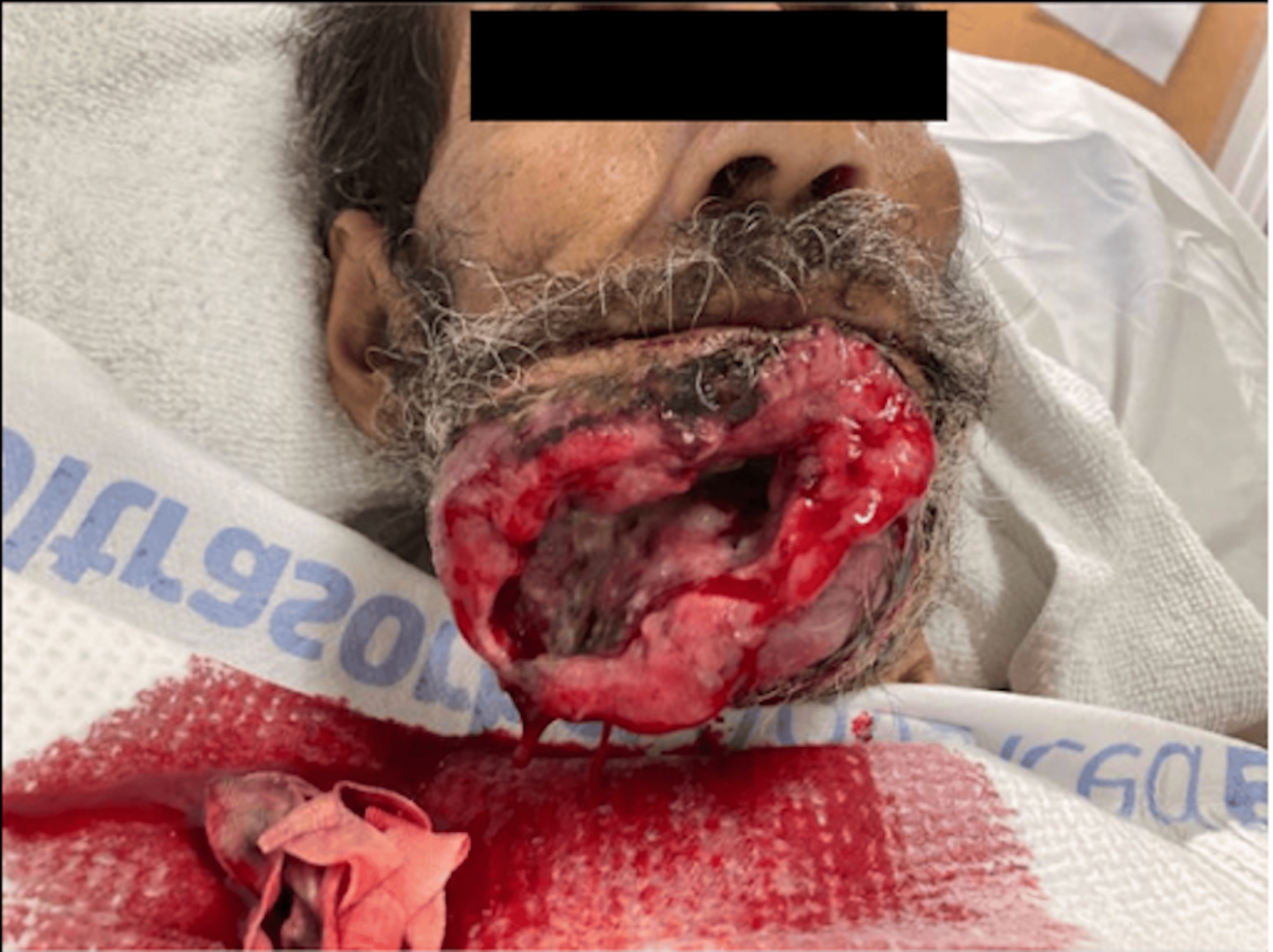
Large orocutaneous fistula with notable erosion of the anterior mandible.

A repeat CT of the soft tissue of the neck was performed during this admission and demonstrated again a large ulcerated, lobulated, peripherally enhancing, and nodular mass involving the mandibular anterior buccal soft tissues extending to the gingival surface and floor of the mouth, concerning malignancy (Figure 2). CT of the chest demonstrated no evidence of acute or metastatic process. On extraoral examination, a large orocutaneous fistula was visualized (Figure 3). Erosion of the anterior mandible with overlying poor dentition and foul drainage was also noted during the inspection. The patient’s tongue was firm, fixed, and restricted in movement on command, leading to dysarthria. The patient endorsed significant pain, which he localized to the site of the orocutaneous fistula. 

Given concerns about inadequate oral intake and failure to thrive, the patient was ultimately admitted. Due to the patient’s lack of oral intake, enteral nutrition was deemed necessary. General surgery was consulted for percutaneous endoscopic gastrostomy (PEG) tube placement; however, the patient instead underwent fluoroscopically guided percutaneous gastrostomy tube placement with interventional radiology to minimize the risk of seeding the known carcinoma during the procedure. Following the establishment of enteral nutrition, the patient was monitored and medically optimized until he was transferred to a dedicated cancer center for planned operative intervention.

**Figure 4:**
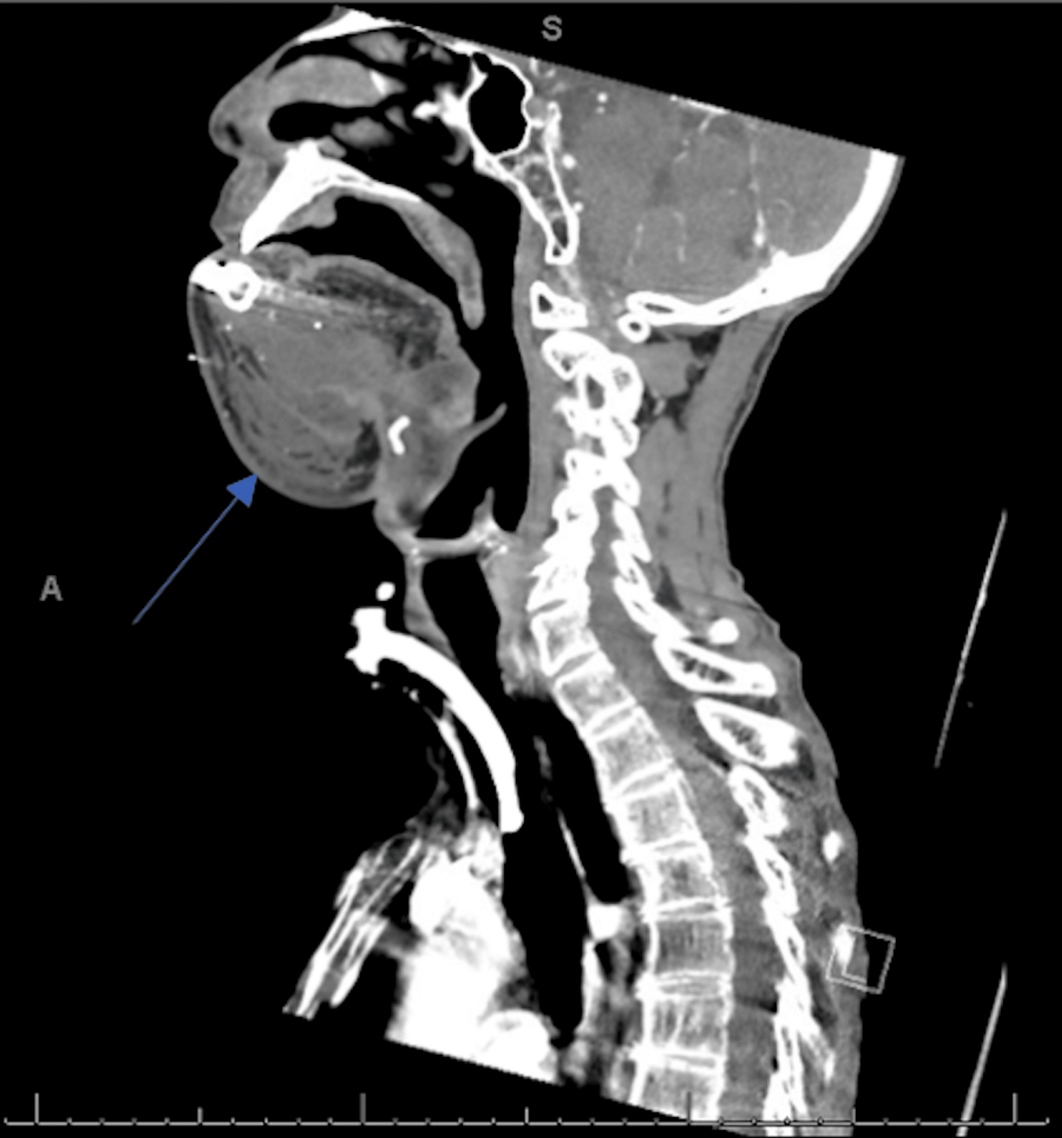
Sagittal view of the neck soft tissue CT status post complex jaw reconstruction with myocutaneous free flap. The scan demonstrates moderate nonspecific swelling of the anterior soft tissues (blue arrow), without definitive evidence of recurrence of malignancy.

Upon transfer, the patient ultimately underwent composite resection of the oral cavity with bilateral modified radical neck dissections, tracheostomy, anterior glossectomy, osteotomy of the neo-mandible and mandibular reconstruction with transosteal plate, left pectoralis major flap with adjacent tissue transfer and Integra placement, right osteocutaneous fibular free flap with Integra placement, and right anterolateral thigh free flap with adjacent tissue transfer. Postoperative imaging demonstrated nonspecific swelling of the anterior soft tissues of the neck status post complex jaw reconstruction with myocutaneous free flap reconstruction without evidence of recurrence of malignancy (Figure 4). The patient tolerated the procedure well and was ultimately discharged with instructions to follow up with oncology regarding future chemoradiation needs.

## Discussion

Oral cancer accounts for approximately 2-4% of all cancer cases worldwide, with OSCC representing more than 90% of all oral neoplasms [2]. The primary sites include the buccal mucosa, upper and lower gingiva, hard palate, tongue, floor of the mouth, and retromolar trigone [4]. Associated risk factors include tobacco and alcohol use, premalignant lesions, increasing age, HIV, and HPV. Survival rates for oral cancer are poor, estimated to be around 50% overall, and no significant improvements have been made in recent decades despite advances in the available therapeutic interventions [5]. Even with the presence of numerous independent prognostic markers for oral cancer, including patient age, tumor location and size, and oncogene expression, the stage of diagnosis remains the most important prognostic factor [4]. Detection at an early stage is the single most effective way to reduce morbidity and mortality, and improve patient quality of life [5].

While prognosis is typically the best when OSCC is diagnosed early, most cases continue to be diagnosed at later stages. Approximately 60% of cases are identified in advanced stages, defined as stages III or IV [4]. Part of the delay in diagnosis is secondary to delayed reporting of symptoms as the initial symptoms experienced by patients tend to be general, including pain, swelling, and other similar complaints of the jaw. Emphasis on awareness and appropriate education of patients is crucial. Providers especially need to be educating patients who have significant risk factors, such as alcohol and tobacco use.

Another aspect to consider in the delay in diagnosis is the role of healthcare providers and the necessity of an appropriate diagnostic workup. In the case report presented above, the patient was previously seen in the emergency department secondary to concerns for facial swelling but was discharged home on antibiotics and the diagnosis was missed. The lack of an appropriate diagnostic workup poses a direct challenge in adequate diagnostic timing and can complicate the future treatment plan. Again, education is crucial to raise awareness for early diagnosis and better prognosis of OSCC. To aid in earlier diagnosis, healthcare providers should be knowledgeable about the early signs of oral carcinoma and should perform oral cancer screening examinations of high-risk individuals, along with educating the general public to raise awareness.

While educational interventions should be provided to the general public, efforts regarding knowledge of disease presentation should be particularly focused on those high-risk patient groups and healthcare professionals. With increased awareness and education regarding the signs and symptoms of OSCC, there is a possibility of earlier diagnosis, improved treatment outcomes, and potentially, higher survival rates.

## Conclusions

In the case described above, the patient initially presented with facial swelling and eventually developed a large orocutaneous fistula before being diagnosed with moderately advanced local OSCC. The patient ultimately underwent complex jaw reconstruction with myocutaneous free flap and is currently working with oncology regarding future chemoradiation needs. Given the correlation of early disease identification with both reduced morbidity and improved patient quality of life, early diagnosis is crucial. Future research efforts should be conducted with an emphasis on improving both prevention and early detection of OSCC.
